# Analysis of Histopathological Findings of Lung Carcinoma in Czech Black Coal Miners in Association with Coal Workers’ Pneumoconiosis

**DOI:** 10.3390/ijerph19020710

**Published:** 2022-01-09

**Authors:** Hana Tomášková, Jaroslav Horáček, Hana Šlachtová, Anna Šplíchalová, Petra Riedlová, Andrea Dalecká, Zdeněk Jirák, Rastislav Maďar

**Affiliations:** 1 Department of Epidemiology and Public Health, Faculty of Medicine, University of Ostrava, Syllabova 19, 703 00 Ostrava, Czech Republic; hana.slachtova@osu.cz (H.Š.); petra.riedlova@osu.cz (P.R.); dalecka.andrea@gmail.com (A.D.); zdenek.jirak@osu.cz (Z.J.); rastislav.madar@osu.cz (R.M.); 2 Public Health Institute Ostrava, Partyzánské Náměstí 7, 702 00 Ostrava, Czech Republic; anna.splichalova@zuova.cz; 3 Institute of Molecular and Clinical Pathology and Medical Genetics, Faculty of Medicine, University of Ostrava, Syllabova 19, 703 00 Ostrava, Czech Republic; Jaroslav.horacek@fno.cz

**Keywords:** lung cancer, histopathological findings, black coal miners

## Abstract

Coal miners with coal workers’ pneumoconiosis (CWP, J60 according to ICD-10) were previously found to have a significantly higher risk of lung carcinoma compared to the general male population. The presented study aimed to analyze the (i) incidence of lung carcinoma in miners, (ii) histopathological findings in cohorts with and without CWP, and (iii) effect of smoking cessation on the histopathological profile. Analyzed cohorts consisted of miners with (*n* = 3476) and without (*n* = 6687) CWP. Data on personal and working history obtained from the medical records were combined with information on lung cancer from the Czech Oncological Register and histopathological findings. Statistical analysis was performed using non-parametric tests and the incidence risk ratio at the significance level of 5%. In 1992–2015, 180 miners (2.7%) without CWP and 169 (4.9%) with CWP, respectively, were diagnosed with lung carcinoma. The risk of lung cancer in miners with CWP was 1.82 (95% CI: 1.48–2.25) times higher than in those without CWP. Squamous cell carcinoma (37%) was the most common histopathological type, followed by adenocarcinoma (22%) and small cell carcinoma (21%). A statistically significant difference between the cohorts (*p* = 0.003) was found in the histopathological subtypes, with the incidence of small cell carcinoma being 2 times higher in miners without CWP than in those with CWP. Only a few individuals with lung carcinoma were non-smokers. The incidence of small cell carcinoma, which is strongly associated with smoking, is significantly higher in miners without CWP. Smoking constitutes the most important risk factor for developing lung carcinoma even in that cohort. However, CWP remains a very important risk factor.

## 1. Introduction

A previous study aimed at the assessment of the risk of developing lung cancer and lung cancer mortality in black coal miners in the Czech Republic (hereafter referred to as miners) revealed a statistically significantly higher risk in miners of black coal who received financial compensation for occupational coal workers’ pneumoconiosis (CWP) compared to the general male population of the Czech Republic [1,2]. Miners without CWP did not have a higher risk than the general male population of the Czech Republic [1,2]. These findings were based on grant projects of the Ministry of Health of the Czech Republic [3,4] and were followed up by the study “Assessment of the carcinogenic risk of coal mine dust” [2]. Based on the results of these studies, a diagnosis of lung cancer in association with pneumoconiosis caused by dust containing free crystalline SiO_2_ was added to the list of occupational diseases in the Czech Republic 2011 [5]. 

The most important coal-mining center in the Czech Republic was concentrated in the areas of Ostrava and Karviná. Before 2000, more than 20,000 miners worked in the mines in this region. In addition to the mining industry, coking, chemical industry, ironworks and other industries were also concentrated in the area, which resulted in the formation of an area considered to have one of the highest environmental burdens in Central Europe [6]. Industrial activity has been declining since the 1990s; nevertheless, the environmental load that has affected the health and lifestyle of the population for several generations still persists. Factors influencing healthy ageing with respect to the environmental load in this area are addressed by the HAIE (Healthy Ageing in Industrial Environment) project [7,8].

Lung cancer is the leading cause of cancer-associated death in men in the Czech Republic, as well as in other countries [9]. Smoking is the principal risk factor of this disease [10]; others include air pollution, occupational factors (e.g., asbestos, silica) or chronic inflammatory diseases, such as chronic obstructive pulmonary disease [11,12,13,14].

The association between smoking and histopathological findings in epidemiological studies has been reported [15]. Lung cancer is typically classified into two major histological types: small cell lung cancer and non-small cell lung cancer; of the latter, adenocarcinoma, squamous cell carcinoma and lung cell carcinoma are the three main subtypes [16]. In smokers, small cell lung carcinoma and squamous cell lung carcinoma predominate while in non-smokers and in women, adenocarcinoma is the most common subtype [15,17,18,19].

As mentioned above, a study investigating the risk of cancer in black coal miners revealed a significantly higher lung cancer mortality (SMR = 1.70; 95% CI: 1.41–2.04) in miners with CWP compared to the general population while in miners without CWP, and the risk was comparable to the general population [2]. The role of smoking in itself is known; however, that of CWP in the development of lung carcinoma remains unclear.

The presented study aimed to analyze (i) the incidence of lung carcinoma in miners relative to the presence of CWP, (ii) the histopathological findings in cohorts with and without CWP, and (iii) the effect of smoking cessation on the histopathological profile.

## 2. Materials and Methods

Analyzed cohorts were miners (CWP cohort) who received reimbursement for occupational CWP between 1992–2013 (3476 miners) and miners without CWP (No-CWP cohort) whose work underground was terminated by 1999 due to reaching more than 90% of the maximum permissible exposure (MPE; exposure to SiO_2_ associated with the 5% probability of miners developing a mild form of CWP; 6687 miners). Both are historical cohorts with continuous follow-ups. The population of the cohorts consists, due to the facts that the population in the Czech Republic is almost exclusively white Caucasian and that coal miners were exclusively males, only of white males.

The CWP cohort data are available from the National Registry of Occupational Diseases (NROD; created in 1992, provision of data was given by a our grant project valid in 2013, hence the cohort recruitment period 1992–2013) [2]. All miners are subject to regular compulsory follow-ups at the occupational health specialist who, if diagnosing CWP, enters the data into NROD, the numbers of CWP patients recruited into the cohort in individual years are shown in the previous paper [2]. The work of workers who develop CWP in mines is automatically terminated and they receive lifelong income as a reimbursement for this problem.

The control group consists of miners whose underground mining work was terminated for preventive reasons, due to reaching 90–100% of the maximal permissible exposure between 1992 and 1999, were alive and did not develop CWP until the evaluation date (2015)—this was verified by their absence in the NROD registry.

Data on participants of both cohorts up to the evaluation date was linked with that from the Czech National Oncological Register (NOR), namely information on lung cancer (C33, C34 according to International Classification of Diseases (ICD)-10 [20]) and histopathological findings (World Health Organization (WHO) classification of tumors of the lung, 2015 [21]). Smoking status was estimated based on the medical records and data in the NOR. Where the information on smoking status was available from both sources but differed, the more serious option was selected (e.g. where one database detailed that the patient was a smoker and the other that he was a non-smoker, the resulting option “smoker” was selected.

For statistical analysis, non-parametric tests (chi-squared test, Fisher’s exact test, Kruskal-Wallis test) were used at the significance level of 5%. In addition, the Incidence risk ratio (IRR) for developing lung carcinoma with 95% confidence interval (CI) was calculated (hence, the parameter Person-years of observation pertains to the lifelong risk of developing lung cancer).

A Kaplan–Meier survival function plot was used for the presentation of the results and a Log-rank test for equality of survival functions for testing the differences between survival functions. All analyses were performed in STATA version 14 (StataCorp LP, College Station, TX, USA) [22] .

The figures of histopathological subtypes of lung cancer presented in this paper originate from preparations of the deceased miners’ lungs and were acquired using an electronic microscope TESTA BS 413 at the Institute of Pathology, University of Ostrava.

### Ethical Consideration

The Ethics Committee of the Institute of Public Health in Ostrava, Czech Republic, approved the study (No. 1/2014).

## 3. Results

### 3.1. Description of the Cohorts

On inclusion into the study, the mean age in the cohort without CWP (No-CWP) was 5.6 years higher than that in the CWP cohort. (Table 1). The mean length of exposure in the mine was approximately 23 years in the No-CWP cohort and 21 years in the CWP cohort, respectively. The information on smoking was available for 98% of patients in the No-CWP cohort and 78% of miners from the CWP cohort. As shown in Table 1, the proportion of non-smokers is identical in both cohorts (33%), but a statistically significant difference (*p* < 0.001) between the proportions of active smokers and ex-smokers between the groups was observed—the proportion of ex-smokers was higher in the CWP than No-CWP cohort.

### 3.2. Lung Cancer

During the period 1992–2015, 180 miners without CWP (2.7% out of the total number of 6687 miners) were diagnosed with lung cancer while in the CWP cohort, 169 individuals (4.9% of the total number of 3476 miners) developed lung cancer. The median age at lung cancer diagnosis in the No-CWP cohort was 63 years (range: 37–79 years), compared to 67 years (range: 44–91 years) in the CWP cohort. A vast majority of lung cancers was detected at late stages due to the absence of a screening program in the Czech Republic at the time, so the cohort is relatively consistent from this perspective. The length of survival from diagnosis (until either death or 2015) was also analyzed. One-year survival was 73% in the No-CWP cohort and 65% in the CWP cohort, respectively. Five-years survival was 6.7% and 7.1% of persons with lung cancer in the No-CWP and CWP cohorts, respectively. No statistically significant difference in survival was found between the cohorts (*p* = 0.262) (Table 2).

**Figure 1:**
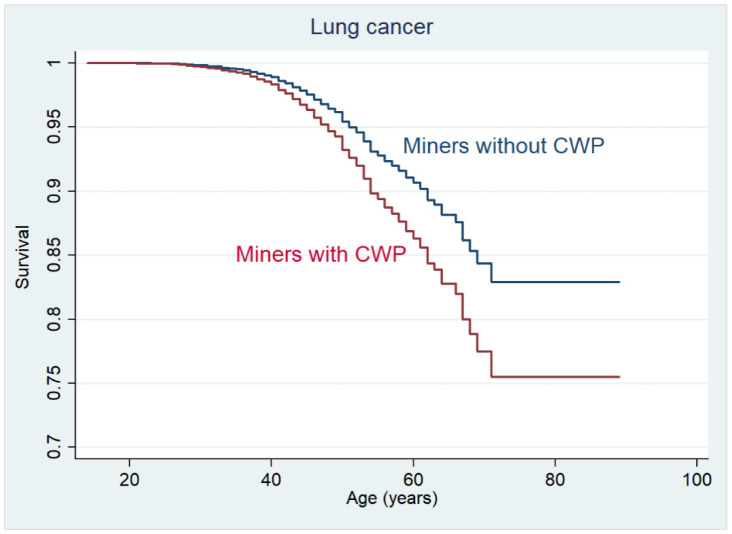
Survival curves for lung cancer-free survival in the cohorts of miners with coal-workers’ pneumoconiosis (CWP) (CWP cohort) and without CWP (No-CWP cohort).

The incidence of lung cancer in the CWP group was approximately 4.24 cases per 10 thousand persons, compared to 7.72 cases per 10 thousand persons in the CWP cohort. The risk of lung cancer in miners with CWP was, therefore, 1.82 times higher (*p* < 0.001) compared to miners without CWP (Table 2). Figure 1 presents the comparison of the cohorts’ survival curves.

### 3.3. Histopathological Findings of Lung Cancer

**Figure 2:**
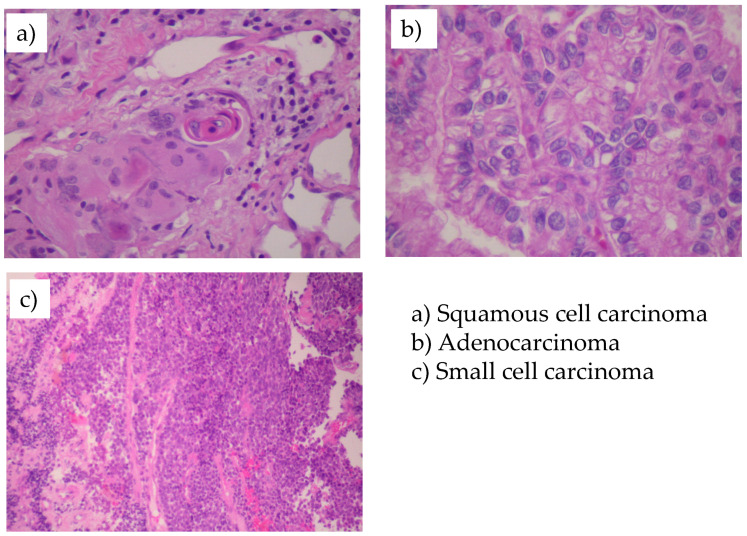
Microscopic photos of histopathological findings from dead miners with lung cancer.

Histopathological findings were available in 79% of patients with lung cancer in the No-CWP cohort and in 81% of patients in the CWP cohort. In all, records on histopathological findings were available in 282 patients with lung cancer (both cohorts combined; 142 in the CWP and 140 in the No-CWP cohorts, respectively). Squamous cell carcinoma was the most common type (37%), followed by adenocarcinoma (23%) and small cell carcinoma (19%). Figure 2 presents histopathological findings from deceased miners.

**Figure 3:**
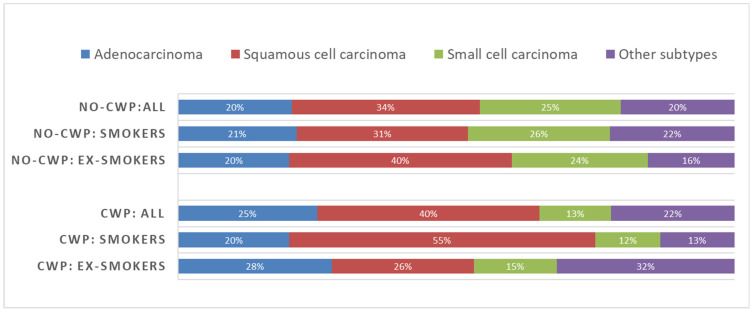
Comparison of histopathological findings of lung cancer in the cohorts of miners according to the presence of coal-workers’ pneumoconiosis (CWP) and smoking status (No-CWP—miners without CWP, CWP—miners with CWP); note that non-smokers were omitted from this figure due to their very small representation in both cohorts (see Table 3).

Squamous cell carcinoma is the most common histopathological subtype in both cohorts (Figure 3). Small cell carcinoma was the second most common subtype in the No-CWP cohort (25%), while in the CWP group, it was found only in 13% of patients. The representation of the histopathological sub-types statistically significantly differed between the cohorts (*p* = 0.003).

The mean age at the time of lung cancer diagnosis varied by the histopathological findings within the range of 61–65 years in the No-CWP cohort and 63–70 years in the CWP cohort. The differences in age at lung cancer diagnosis relative to the histopathological findings were not statistically significant within cohorts (*p* = 0.3776 and *p* = 0.111, respectively) but the mean age at the time of lung cancer diagnosis significantly differed between the CWP and No-CWP cohorts (*p* < 0.001), being higher in the CWP cohort (Table 3).

In the CWP cohort, a statistically significant association between the length of survival and histopathological findings was detected (*p* = 0.002), with median survival lengths ranging from 0.2 to 0.7 years. The shortest survival was detected in the subgroups with small cell carcinoma and adenocarcinoma. On the contrary, there was no statistically significant difference in survival length according to histopathological findings (*p* = 0.182, see Table 3). No statistically significant difference was found between cohorts, either (*p* = 0.770).

### 3.4. Smoking and Lung Cancer

Records on smoking status were missing in 18% (*n* = 30) of the patients with lung cancer within the CWP cohort while it was available for all patients with lung cancer within the No-CWP cohort. There was a statistically significant difference in smoking status in miners with lung cancer between the cohorts (*p* < 0.001). The proportion of ex-smokers and non-smokers was higher in the CWP than the No-CWP cohort; in the No-CWP cohort, active smokers predominated (Table 2).

Interestingly, only 1.7% (No-CWP) and 10.8% (CWP) of non-smokers were in the cohorts with lung carcinoma while in the total cohorts, 33% were non-smokers (Table 1), which indicates the importance of smoking as an etiological factor.

### 3.5. Histopathological Findings of Lung Cancer and Smoking Status

Data on histopathological findings were available for 142 out of 180 miners with lung carcinoma in the No-CWP cohort and for 116 out of 139 miners (Table 3) in the CWP cohort. In the No-CWP cohort, no statistically significant association between the smoking status (smokers/ex-smokers) and histopathological classification was detected (*p* = 0.882). Squamous cell carcinoma and small cell carcinoma were the most common histopathological types in this group. It is necessary to point out that only three patients in this cohort with lung carcinoma were non-smokers. In the CWP cohort, however, a statistically significant association of the smoking status with histopathological findings was revealed (*p* = 0.007). Squamous cell carcinoma was the most common subtype in active smokers (55%) followed by adenocarcinoma (20%) and small cell carcinoma (12%). In ex-smokers, other histopathological types represented the most common histopathological findings (32%), while the shares of squamous cell carcinoma and adenocarcinoma were similar (26% and 28%, respectively). Small cell carcinoma was present in 15% of ex-smokers in the CWP cohort with lung cancer (Table 3, Figure 3).

## 4. Discussion

The risk of lung cancer was statistically significantly higher in miners with CWP compared to miners without CWP (IRR = 1.82), which is consistent with the results of previous analyses [1,2,23].

The representation of non-smokers was approximately 33% in both cohorts, but in miners with lung cancer, the incidence of ex-smokers was very low (No-CWP—2%, CWP—9%). The proportion of ex-smokers was higher in the CWP cohort than in the non-CWP cohort, which may be explained by pre-existing health problems and the CWP diagnosis [2].

In the No-CWP cohort, 180 individuals (2.7%) were diagnosed with lung carcinoma by 2015; this number was higher in the CWP group (169 individuals, i.e., 4.9%). The mean age at diagnosis was approximately 63 years in the No-CWP group and 67 years in the CWP group. Compared to the general male population of the Czech Republic, lung carcinoma was diagnosed at a younger age in both cohorts—the median age at diagnosis in Czech males is 69 years of age (IQR: 63–74) [24]. We must, however, consider that the cohorts in this study are living, which also contributes towards the younger mean age at diagnosis.

The five-year survival in patients with lung carcinoma in the general population in the Czech Republic was approximately 13% in 2004–2008, which grew to 17.8% between 2014 and 2018 [24,25]. In both our cohorts, however, five-year survival was only approximately 7%. Such a low percentage could be caused by various factors, such as the lifestyle and generally extremely demanding working conditions in the mines, which can also affect the general condition of the miners; this can, in turn, lead to underestimation of new symptoms and, thus, to the diagnosis of lung carcinoma at later stages. The data we had, however, did not contain staging at diagnosis; nevertheless, the data available for the general population in 2014–2018 indicate that most lung tumorous were diagnosed at stages III and IV, which are associated with shorter survival (about 3% for Stage IV and 12% for Stage III). Five-year survival for Stage II is over 20% and for Stage I, it is more than 40% [24].

Morphological types of lung cancer are generally classified into small cell and non-small cell lung carcinomas, with the latter including mainly adenocarcinoma and squamous cell carcinoma. Until the 1990s, squamous cell carcinoma was the predominant type of lung cancer both in the Czech Republic [24] and worldwide [24,26,27], followed by adenocarcinoma, which is more common in women [26]. Smoking is considered the main risk factor for lung carcinoma, being associated with its occurrence in approximately 90% of men and 60–80% of women [10,28]. Since the 1990s, a worldwide decline in smoking has been reported worldwide, especially in men. Besides, the cigarette composition and designs have also changed [25,28,29], which may be associated with the gradual change in the morphological types of smoking-associated lung cancer, i.e., with the growing representation of adenocarcinoma at the expense of squamous cell carcinoma [25,26,27,30,31]. Squamous cell carcinoma was the most common histological type of lung cancer (approximately 30 % of cases), followed by adenocarcinoma (23 %) and small cell carcinoma (14 %) in males in the general population of the Czech Republic between 1994 and 1998, while from 2014–2018, their representation evened out—squamous cell carcinoma was detected in 25% and adenocarcinoma in 26% of patients.

The representation of individual histopathological findings differs with sex. In 2014–2018, squamous cell carcinoma was the most common type (approximately 30% of cases), followed by adenocarcinoma (23%) and small cell carcinoma (14%) in males in the general population of the Czech Republic [24,32]. Compared to that, the CWP cohort had a higher representation of squamous cell carcinoma (40%) with comparable proportions of adenocarcinoma and small cell carcinoma. In the No-CWP cohort, we detected a higher representation of small cell carcinoma (25%) compared both to the general male Czech population and the CWP cohort. To compare it with data from abroad, we can refer to a study from the USA [27] reporting the occurrence of small cell carcinoma in males in 2004–2009 (9.8%), which showed it as being lower than in any of our cohorts as well as than in the general Czech male population. The occurrence of squamous cell carcinoma was also lower in the USA (18.8%) than in the Czech Republic, while the incidence of adenocarcinoma was comparable (24.5%).

Small cell carcinoma predominantly occurs in smokers, mostly in heavy smokers and only very rarely in non-smokers [15,19,33]. A Korean study reported the proportion of smokers (including ex-smokers) in males with small cell carcinoma to be 89.3% (of which 10% were ex-smokers), with similar numbers for squamous cell carcinoma (88.3%, of which 11.7% were ex-smokers). The lowest representation of smokers was observed in adenocarcinoma (72.9%, of which 14.9% were ex-smokers) [18]. Similar results were reported also by Kollarová et al. [17], with the risk estimates of individual histopathological subtypes relative to smoking being higher for squamous cell and small cell lung carcinoma (OR = 17.9 and OR = 18.6, respectively) than for adenocarcinoma (OR = 7.4). Based on the analysis of the SYNERGY database [28], odds ratios of “current male smokers with an average daily dose of >30 cigarettes” were 103.5 (95% CI: 74.8–143.2) for squamous cell lung carcinoma, 111.3 (95% CI: 69.8–177.5) for small cell lung carcinoma and 21.9 (95% CI: 16.6–29.0) for adenocarcinoma. Smoking cessation reduced the relative risk in the short term and long term, but risks among heavy former smokers never fully returned to baseline risks of non-smokers [28].

The greatest difference in the representation of histopathological types of lung carcinoma between CWP and No-CWP cohorts was found for the small cell carcinoma, which was almost twice as high in the No-CWP than in the CWP group; the above-mentioned studies imply that small cell lung carcinoma is more strongly associated with smoking than the other histopathological subtypes, particularly adenocarcinoma. The representation of adenocarcinoma was higher in the CWP cohort. Both of these findings correlate with the significant difference in the representation of smokers and ex-smokers in the CWP and No- CWP cohorts, respectively, which is (as mentioned above) probably caused by the fact that patients with CWP are more likely to stop smoking due to their condition.

Squamous cell carcinoma was the most common histological subtype in both cohorts. Squamous cell carcinoma is also strongly associated with smoking, but its occurrence in smokers was reported to be significantly associated also with chronic obstructive pulmonary disease [10,13]. As CWP and COPD are both diseases based on chronic inflammatory processes in the lungs, and as chronic inflammatory processes as such are known to constitute risk factors for tumour development [34,35], it is not surprising that the same is true for smokers in the CWP cohort.

### Limitations

The limitations of the presented study include incomplete records on smoking status. In miners with CWP, this information was available for the vast majority of patients, which is due to the recording of this data by the occupational health clinics. Still, these records did not contain detailed information on smoking—data on the smoking duration and daily cigarette consumption were missing; in former smokers, neither the time (how long ago) of smoking cessation nor the reason for it were recorded. Data on miners with CWP were also available from the Czech National Cancer Registry; however, these were incomplete as well. Another limitation concerns the Czech National Cancer Registry [8]. The data on the histopathological findings were not recorded for all miners; they were available only for 78% of miners without CWP and 82% of miners with CWP. Besides, the so-called "healthy worker effect” [2] and age at inclusion in the study can be considered another limitation. Although miners with CWP are older (mean difference of 5.6 years), we must take into account that both cohorts are still living and are relatively young. They will be observed further.

## 5. Conclusions

We confirmed a higher risk of lung cancer development in miners with CWP than in those without CWP, with a relative risk of approximately 1.82.

From the perspective of histopathological findings, squamous cell carcinoma was the most common (40% in the No-CWP and 34% in the CWP cohort, respectively). In miners with CWP, adenocarcinoma was the second most common, but in the cohort without CWP, small cell carcinoma, which is significantly associated with smoking, was the most common. The occurrence of small cell carcinoma in the CWP cohort is approximately half of that in the No-CWP cohort. The difference in proportions of histopathological findings is significant.

Representation of histopathological findings did not differ between smokers and ex-smokers in the No-CWP cohort. On the other hand, it was statistically significant in the CWP cohort, especially where squamous cell carcinoma is concerned; this type occurred in 55% of smokers and only 26% of ex-smokers. In both these groups, small cell carcinomas accounted for less than 15 % of lung cancers.

Still, despite the limitations of the study, we can confirm that smoking is the principal risk factor in the No-CWP cohort. In miners with CWP, however, the disease counts among significant risk factors, leading to an almost two-fold increase in the risk of development of lung cancer compared to the miners without CWP.

## Figures and Tables

**Table 1:** Description of the groups of miners without/with coal-workers’ pneumoconiosis.

Characteristics of Cohorts		No-CWP (*n* = 6687)	CWP (*n* = 3476)
Age at study entry (years) (mean ± SD)		44.0 ± 6.3	49.6 ± 12.4
Exposure in a mine (years) (mean ± SD)		22.9 ± 5.9	20.7 ± 7.8
Smoking habits *	smokers	54%	45%
	ex-smokers	13%	22%
	non-smokers	33%	33%
Chi-squared test		*p* < 0.001	

* smoking status recorded for 98% of individuals in the No-CWP cohort and 78% in the CWP cohort, respectively, CWP—coal-workers’ pneumoconiosis, SD—Standard deviation.

**Table 2:** Description of miners without/with coal-workers’ pneumoconiosis (CWP) and lung cancer.

Cohort		No-CWP (CWP-0) (*n* = 6687)	CWP (CWP-1) (*n* = 3476)
Lung cancer *n* (%)		180 (2.7%)	169 (4.9)
Age at lung cancer diagnosis (mean ± SD)		62.6 ± 7.6	66.6 ± 9.5
Length of survival *	<1 year	131 (72.8%)	110 (65.1%)
	1–5 years	37 (20.5%)	47 (27.8%)
	≥5 years	12 (6.7%)	12 (7.1%)
Chi-squared test		*p* = 0.262	
Person-years of observation *		424,499	218,834
IR per 10,000 person-years (95% CI)		4.24 (3.65–4.90)	7.72 (6.62–8.96)
IRR		1.82 (1.48–2.25)	
Smoking	Active smokers	121 (67.2%)	69 (49.6%)
	Ex-smokers	56 (31.1%)	39 (39.6%)
	Non-smokers	3 (1.7%)	15 (10.8%)
	Total	180 (100%)	139 (100%)
Chi-squared test		*p* < 0.001	

No-CWP cohort—miners without CWP; CWP cohort—miners with CWP; CI—Confidence Interval; IR—Incidence Rate, IRR—Incidence Rate Ratio; * from birth to lung carcinoma diagnosis, death or 2015, SD—Standard deviation.

**Table 3:** Age at the time of the diagnosis, length of survival and distribution of smoking status classified by histopathological findings of lung.

Cohort	Lung Cancer		Age at the Time of Diagnosis			Length of Survival *			Smoking Status		
	Histopathological Findings	*N* (%)	Median	Mean (±SD)	Min.–Max.	Median	Mean (±SD)	Min.–Max.	Active	Ex-Smokers	Non-Smokers
No-CWP	Adenocarcinoma	29 (16%)	64.0	61.9 ± 7.67	37–72	0.4	0.9 ± 1.25	0–5.9	20 (21%)	9 (20%)	0 (0%)
	Squamous cell carcinoma	48 (27%)	62.0	62.4 ± 7.49	46–76	0.6	1.5 ± 2.04	0–7.9	29 (31%)	18 (40%)	1 (33%)
	Small cell carcinoma	36 (20%)	62.5	61.8 ± 5.86	51–76	0.3	1.0 ± 2.04	0–11.4	24 (26%)	11 (24%)	1 (33%)
	Other subtypes	29 (16%)	63.0	61.0 ± 8.01	46–73	0.7	3.5 ± 6.64	0–21.5	21 (22%)	7 (16%)	1 (33%)
	Missing	38 (21%)	65.5	64.5 ± 8.69	43–79	0.2	0.6 ± 1.31	0–7.9	-	-	-
	Total	180 (100%)	63.0	62.4 ± 7.59	37–79	0.4	1.4 ± 3.22	0–21.5	94 (100%)	45 (100%)	3 (100%)
	Kruskal-Wallis test/Fisher’s exact test		*p* = 0.3776			*p* = 0.0024			*p* = 0.882		
CWP	Adenocarcinoma	35 (21%)	64.0	63.0 ± 8.54	44–78	0.3	1.5 ± 3.28	0–17.9	12 (20%)	13 (28%)	2 (22%)
	Squamous cell carcinoma	56 (33%)	68.0	66.1 ± 9.27	46–87	0.9	1.4 ± 2.02	0–9.7	33 (55%)	12 (26%)	1 (11%)
	Small cell carcinoma	18 (11%)	68.5	66.3 ± 9.04	47–81	0.5	2.0 ± 4.41	0–17.5	7 (12%)	7 (15%)	1 (11%)
	Other subtypes	31 (18%)	69.0	69.7 ± 9.52	48–89	0.5	2.1 ± 4.49	0–19.5	8 (13%)	15 (32%)	5 (56%)
	Missing	29 (17%)	70.0	68.6 ± 10.41	48–91	0.2	1.0 ± 1.98	0–9.8	-	-	-
	Total	169 (100%)	67.0	66.6 ± 9.52	44–91	0.5	1.5 ± 3.14	0–19.5	60 (100%)	47 (100%)	9 (100%)
	Kruskal-Wallis test/Fisher’s exact test		*p* = 0.111/*p* < 0.001 **			*p* = 0.1817/*p* = 0.770 **			*p* = 0.007		

* to death or year 2015, ** comparison between cohorts,—Not applicable, SD—Standard deviation.

## Data Availability

Data are available from the authors upon reasonable request.
